# Educación virtual en odontología durante la pandemia de COVID-19

**DOI:** 10.21142/2523-2754-0903-2021-078

**Published:** 2021-10-06

**Authors:** Darlyng Nicolle Guevara-Veliz, Keyla Margorin Flores-Joaquin, Alex Gianmarco Maturrano-Santos, Manuel Antonio Mattos-Vela

**Affiliations:** 1 Facultad de Odontología, Universidad Nacional Mayor de San Marcos. Lima, Perú. darlyngguevarav@gmail.com, keyla.flores@unmsm.edu.pe, alex.maturrano@unmsm.edu.pe Universidad Nacional Mayor de San Marcos Facultad de Odontología Universidad Nacional Mayor de San Marcos Lima Peru darlyngguevarav@gmail.com keyla.flores@unmsm.edu.pe alex.maturrano@unmsm.edu.pe; 2 Departamento Académico de Estomatología Biosocial, Facultad de Odontología, Universidad Nacional Mayor de San Marcos. Lima, Perú. mmattosv@unmsm.edu.pe Universidad Nacional Mayor de San Marcos Departamento Académico de Estomatología Biosocial Facultad de Odontología Universidad Nacional Mayor de San Marcos Lima Peru mmattosv@unmsm.edu.pe

**Keywords:** educación en odontología, educación a distancia, infecciones por coronavirus, realidad virtual, education dental, education distance, COVID-19, virtual reality

## Abstract

Los cambios en la educación universitaria que surgieron en el contexto de la pandemia de COVID-19 supusieron nuevos retos en todo el mundo. En el caso de la educación odontológica, si bien ya se había intentado la implementación de recursos virtuales, un enfoque completamente virtual fue un nuevo desafío. El objetivo de este artículo es discutir sobre la educación virtual en el área odontológica en el contexto de la pandemia de COVID-19 y los retos que representa. Se concluyó que, para lograr la continuación de la educación dental en una modalidad virtual, las estrategias más usadas por muchas universidades fueron las plataformas académicas Moodle y, para realizar las videoconferencias, Google Meet y Zoom. Asimismo, la percepción de esta nueva modalidad, tanto de docentes y estudiantes en su mayoría fue buena; sin embargo, se enfatiza que estas no reemplazan la necesidad de las actividades presenciales para desarrollar sus capacidades como futuros odontólogos.

## Abstract (en)

The changes in university education that appeared within the context of the COVID-19 pandemic posed new challenges throughout the world. In the case of dental education, although the implementation of virtual resources had already been tried, a completely virtual approach was a new challenge. The objective of this article is to discuss virtual education in the dental area within the context of the COVID-19 pandemic and the challenges it represents. In conclusion, for the continuation of dental education in a virtual modality, the strategies most used by many universities are the academic platforms Moodle and for videoconferencing, Google Meet and Zoom. Likewise, the perception of this new modality, both by teachers and students, was mostly good; however, it is emphasized that these methods cannot replace the need for face-to-face activities for students to develop their capacities as future dentists.

## INTRODUCCIÓN

Con el aumento de casos de contagio por COVID-19 y, posteriormente, el anuncio del confinamiento en varios países, las universidades tuvieron que implementar el uso de estrategias virtuales para continuar con la educación superior en un nuevo modelo no presencial ^1^. Si bien las plataformas digitales, conferencias virtuales y seminarios web ya han sido usados anteriormente en cursos de Odontología en modalidades semipresenciales o como recurso complementario, el contexto actual supuso una modalidad virtual en su totalidad ^2^^,^^3^. Así, las facultades tuvieron que adaptar el plan curricular para que los estudiantes fueran capaces de lograr las competencias requeridas, y usaron para ello las plataformas disponibles. 

No se puede negar que esta pandemia está generando nuevas estrategias para mantener la calidad de la educación odontológica, muchos de los cuales podrían ser adaptados e incluidos en la enseñanza cotidiana en las facultades en un futuro. Por ello, es necesario reflexionar sobre los resultados de estudios realizados sobre la educación virtual llevadas a cabo durante este periodo. El propósito de este trabajo es discutir sobre la educación virtual y los retos que representa para la odontología en el contexto de la pandemia de COVID-19.

## ANTECEDENTES DE LA VIRTUALIDAD EN ODONTOLOGÍA

En el 2008, el consejo de Europa aprueba la declaración con la cual se respalda la efectividad del aprendizaje virtual para impulsar la equidad y el desarrollo educativo en la Unión Europea. Luego de ello, ascendería el *boom* de los MOOC (*Massive Open Online Course*), los cuales se establecen con el objetivo de ofrecer conocimiento y educación en línea al alcance de cualquier persona con acceso a internet y dispositivos electrónicos vinculados con el mismo; en paralelo, surge la revolución industrial y la eliminación de la centralización del conocimiento, con lo cual el entorno facilita la accesibilidad a la población ^4^.

Es por ello, que en la última década, el desarrollo de las nuevas tecnologías de la información y la comunicación (TIC) se incrementó considerablemente en la práctica de todas las universidades reconocidas en el mundo, las cuales ofrecen programas de educación virtual. La comunidad odontológica no fue ajena a este desarrollo tecnológico ^5^.

En 1958, el Dr. Robert Ledley, dentista de la ciudad de Nueva York (EE. UU.) utilizó por primera vez una computadora para el análisis de pacientes; a partir de ese momento, se estableció un hito en la historia de la odontología, se obtuvieron programas y tecnologías que permitieron la historia clínica computarizada, el diagnóstico automatizado de caries, el desarrollo de áreas de bioinformática, entre muchas otras herramientas y tecnologías de información que han utilizado en los últimos años las distintas entidades e instituciones para cumplir con los objetivos educativos en la implementación de la virtualidad ^6^. 

La adquisición de tecnologías digitales en los planes curriculares de estudio en la carrera de Odontología se implementó a nivel mundial, y alcanzó diferentes niveles de abordaje según la demanda de los estudiantes y docentes. Se han introducido programas de educación 3D para perfeccionar la capacidad de determinar espacio-tiempo en estudiantes y conocer la morfología dental y la planificación de tratamientos ^7^. Al establecer una enseñanza virtual, se estima un sistema de gestión del aprendizaje que incluye una combinación de herramientas, instalaciones y TIC como ruta de aprendizaje; dentro de ellos, cuestionarios, tareas semanales, enlaces útiles, artículos relacionados e interacciones activas ^8^.

Asimismo, en varios países ya se había comenzado a implementar las TIC, principalmente como complemento de las actividades presenciales realizadas normalmente en las facultades de Odontología. La Tabla 1 presenta investigaciones realizadas para conocer la respuesta de los estudiantes a esta nueva modalidad, la cual es mayoritariamente positiva y resalta que los recursos virtuales ahorran tiempo y mejoran la comunicación con el docente y otros estudiantes. 

**Tabla 1:** Artículos originales sobre implementación de la virtualidad publicados antes de la pandemia

AUTOR	OBJETIVO	TIC	RESULTADOS
Moshabab, Arabia Saudita, 2017	Identificar la preparación de los estudiantes para el aprendizaje online, investigar su percepción y medir la calidad de las tutorías online	AP* + conferencias online, videos, uso de Twitter y Google Moderator (comunicación de docentes y estudiantes)	La mayoría de los estudiantes respondieron positivamente con respecto a la aceptabilidad y usabilidad del aprendizaje en línea.
Cantarini et al., Argentina, 2017	Analizar la influencia de las TIC en la enseñanza de las asignaturas de la carrera de Odontología	AP* + plataforma Moodle	Facilitó la comunicación, la participación y la realización de tareas y evaluaciones.
Castro et al., Perú, 2017	Evaluar la percepción de los estudiantes de posgrado sobre la implementación del enfoque b-learning como metodología para el proceso enseñanza-aprendizaje	AP* + plataforma de Facebook	La mayoría de los estudiantes se sintieron satisfechos con el enfoque presencial-virtual.
Inquimbert et al., Francia, 2019	Identificar el tipo de apoyo pedagógico que los estudiantes prefirieron en cada curso y. luego. evaluar el impacto que tuvo este nuevo formato pedagógico	Video que describe las diferentes etapas del tratamiento de conductos	Todos los estudiantes se sintieron satisfechos por la naturaleza visual y concisa del video.
Soltanimehr et al., Irán, 2019	Comparar el efecto de la educación virtual y tradicional en el conocimiento teórico y en la presentación de informes de los estudiantes de Odontología en la interpretación radiográfica de lesiones óseas de la mandíbula	AP* + sistema de gestión del aprendizaje (presentaciones en Power Point, libros y artículos en formato PDF, y enlaces a otros sitios web educativos)	La enseñanza teórico-práctica tuvo un efecto significativo en la evaluación teórica, pero no en la evaluación clínica.

AP*: Actividades presenciales

## ESTRATEGIAS PARA LA EDUCACIÓN VIRTUAL EN ODONTOLOGÍA

Aparecen nuevas metodologías de enseñanza que ayudan a la edificación del aprendizaje y amplían las alternativas de conocimiento en lo que conocemos como educación virtual; pero, con ello, los expertos han buscado nuevos recursos que le permitan a esta nueva forma de aprendizaje cumplir los objetivos de una educación tradicional y contribuir a la calidad educativa ^9^^-^^11^.

Se han establecido sistemas de realidad virtual en universidades del Reino Unido, lo cual permite afrontar las limitaciones con las que se cuenta en la educación virtual, así como también las TIC, las cuales permiten administrar información, datos y conocimiento que se transfieren a los estudiantes para el óptimo desarrollo de competencias ^2^^,^^5^. Así mismo, en la comunidad hispana se ha instalado una metodología didáctica que ayuda al estudiante con el fin de integrar conocimientos, que consiste en el aprendizaje colaborativo (AC), metodología por la cual los estudiantes trabajan de manera integral y coordinada, como agentes activos y creativos en las tareas asignadas. La estrategia consiste en integrar el AC con el uso de las TIC desde dispositivos electrónicos, con el fin de desarrollar clases más dinámicas y atractivas ^12^. 

Por otro lado, los avances en las ciencias médicas y odontológicas en estas últimas décadas han sido muy significativos, por lo que el sistema educativo tradicional resulta insuficiente. Por esa razón, se siguen buscando estrategias, como plataformas virtuales sostenibles que alberguen información y, al mismo tiempo, sean un lugar de interacción entre docentes y estudiantes, con tutorías web de fácil accesibilidad y uso, así como con la posibilidad de repetir la práctica. De acuerdo con la naturaleza del contenido educativo, se incluyeron películas educativas de procedimientos dentales, acceso a libros de resúmenes de congresos, discusiones relacionadas a casos clínicos, galerías de fotos y programas de aprendizaje electrónico ^13^.

El enfoque educativo denominado *blended learning* (*b-learning*) tiene como fin mejorar los programas de enseñanza-aprendizaje integrando las fortalezas del aprendizaje tradicional (sincrónico) con actividades en línea (asincrónicas) para beneficiar la motivación y el compromiso del estudiante de Odontología con el aprendizaje y la interacción. Esta metodología nutre el método formativo, al individualizar la educación y cubrir muchos más objetivos de aprendizaje ^14^, es decir, se está conectando la educación tradicional y la educación virtual aplicando la Odontología basada en evidencia. Otra estrategia utilizada por excelencia para educar en la responsabilidad, que ayuda al estudiante valorar, criticar y reflexionar sobre su proceso de aprendizaje, es la autoevaluación, pues, dentro de los límites que presenta la educación virtual, ayudará a consolidar las metodologías y estrategias planteadas en esta realidad ^15^.

## PERCEPCIÓN DE LOS DOCENTES Y ESTUDIANTES RESPECTO DEL USO DE LA VIRTUALIDAD EN EL CONTEXTO DE LA PANDEMIA DE COVID-19

La educación online conecta profesores y estudiantes, lo que la hace más accesible para todos y brinda una opción diferente al aprendizaje presencial, pero también tiene desventajas que afectan a ambos grupos, como la falta de acceso a recursos tecnológicos (ordenador, laptop, *smartphones*, *tablets*, entre otros) y una conexión a internet. Esto ha supuesto uno de los principales problemas durante el confinamiento, ya que la educación ha quedado subordinada a la virtualidad, y ha puesto en evidencia la desigualdad socioeducativa y la brecha digital existente, mayoritariamente, en varios países subdesarrollados. Por otra parte, la sensación de sentirse solo y tener problemas de adaptación a la nueva modalidad es un problema que ha sido recurrente, por lo que se le están dando importancia a la salud mental de los estudiantes en el contexto de pandemia ^16^.

La consideración con respecto a los entornos virtuales de aprendizaje presenta polaridad frente a distintas herramientas tecnológicas, es decir, algunas como Moodle, Google Classroom, Google Meet y Zoom presentan una alta valoración, pues los estudiantes mencionan que son instrumentos facilitadores de intercambio de información entre docentes y estudiantes ^17^; sin embargo, señalan que se deben complementar con prácticas no virtuales.

Así, en los distintos artículos revisados, se observó una alta aceptación del enfoque totalmente virtual y los beneficios en su rendimiento académico; sin embargo, también se evidenció la necesidad de actividades presenciales, ya que los estudiantes no consideran que puedan ser reemplazadas por la virtualidad ^18^^,^^19^. También se sugirió que, después de la pandemia, se combinen los componentes tanto presenciales como en línea con el fin de mejorar la educación dental.

En el caso de los docentes, a pesar de que los contenidos académicos no cambian, la virtualidad influye en la calidad de enseñanza y la puede volver un poco compleja; por ende, deben adaptar su enseñanza a las necesidades de aprendizaje de los estudiantes ^20^. En el pasado, los docentes usaban herramientas como los libros de texto, folletos, entre otros, pero actualmente las plataformas virtuales están ganando popularidad en la enseñanza odontológica ^21^.

En el caso de los estudiantes de posgrado, también es una alternativa para el proceso de enseñanza, el aprendizaje y su respectiva evaluación, porque implica un ahorro de tiempo con respecto a las actividades presenciales y eso los ayuda a lograr sus objetivos en cada materia ^14^.

## NUEVOS RETOS DE LA EDUCACIÓN ODONTOLÓGICA DURANTE LA PANDEMIA DE COVID-19

La pandemia de COVID-19 ha generado nuevos retos para las personas a nivel mundial. El mayor desafío para la administración de las escuelas de Odontología es tratar de equilibrar la importante tarea de cuidar la salud de los estudiantes y, al mismo tiempo, asegurar la continuidad de la educación ^22^. Por ello, han surgido propuestas de solución para reanudar la educación odontológica de manera virtual, como el uso de las diferentes plataformas para el dictado de clases, la evaluación y los exámenes ^23^. Además, el aprendizaje para la parte clínica también se puede realizar de manera virtual, a través de dispositivos de simulación en realidad virtual que son útiles para el desarrollo de las habilidades manuales ^24^^-^^26^.

Los beneficios de una enseñanza virtual son el acceso a la navegación por internet y la diversidad de formas de enseñanza (videoconferencias, foros, consultas, seminarios, entre otros) que serán usados para mejorar el aprendizaje tradicional y evitar la pérdida de clases universitarias debido a la suspensión de las actividades presenciales ^27^. La educación estomatológica tendrá muchos cambios de gran repercusión, por ejemplo, la inversión en educación virtual moderna de fácil acceso para docentes y estudiantes, el compromiso de los docentes para mantenerse motivados y seguir aprendiendo nuevas herramientas virtuales que mejoren los métodos de enseñanza tradicionales y el compromiso de las casas de estudio para capacitar a sus docentes, no solo en educación virtual, sino también en la enseñanza profesional, porque un buen docente podrá elegir el mejor método, las mejores estrategias y las mejores herramientas para llegar a sus estudiantes ^1^^,^^28^.

En el marco de la contingencia producida por la pandemia de COVID-19, debemos reconocer que los recursos tecnológicos, como los entornos virtuales, dan la oportunidad de reinventar la educación superior y adaptarla a la condición no presencial que exige el momento actual ^29^^,^^30^.

## CONCLUSIONES

La educación virtual se ha convertido en la solución actual para continuar con la enseñanza de miles de universitarios debido al confinamiento por la pandemia del COVID-19. Se destaca que la virtualidad se había introducido anteriormente en diversos países y universidades con la modalidad de los MOOC y, posteriormente, como las TIC para mejorar la enseñanza de los cursos. En el caso de la carrera de Odontología, se habían implementado con el fin de mejorar la enseñanza-aprendizaje, además de que la mayoría de los estudiantes opinaba que tenían un efecto positivo en los cursos, sin embargo, las TIC se limitaban a complementar las actividades presenciales y no como una modalidad totalmente virtual.

Tras casi un año desde el inicio de clases virtuales en varias facultades de Odontología, se escribieron varios artículos sobre la percepción de docentes y estudiantes respecto de los cursos virtuales, generalmente positiva, pero que reconoce las limitaciones tanto para el estudiante como el docente. El problema más evidente para ambos fue el acceso a los recursos tecnológicos (ordenador, *laptop*, celular inteligente, *tablets*, entre otros) y la conexión a internet. Sin embargo, el contexto de la pandemia ha traído soluciones para enfrentar la interrupción de clases presenciales, generalmente, fue indispensable el uso de plataformas académicas como Moodle y, para videollamadas, el uso de Google Meet y Zoom, así como las plataformas propias de cada universidad. La inclusión de estas herramientas tecnológicas abre la puerta a un mundo de oportunidades y retos en el futuro más cercano para los estudiantes de Odontología, como el aprendizaje virtual usando dispositivos de simulación en realidad virtual, útiles para el desarrollo de las habilidades manuales que necesita un futuro odontólogo.

## Fuente de financiamiento:

Financiado por los autores.
